# Whole-Genome Sequence of CMY-2 β-Lactamase-Producing *Salmonella enterica* Serovar Typhimurium Strain L-3553

**DOI:** 10.1128/genomeA.00711-14

**Published:** 2014-07-24

**Authors:** Tsuyoshi Sekizuka, Ken-ichi Lee, Makoto Kuroda, Masahiro Kusumoto, Taketoshi Iwata, Ikuo Uchida, Kiyoshi Tanaka, Yukino Tamamura, Masato Akiba

**Affiliations:** a Pathogen Genomics Center, National Institute of Infectious Diseases, Shinjuku, Tokyo, Japan; b Bacterial and Parasitic Disease Research Division, National Institute of Animal Health, National Agriculture and Food Research Organization, Tsukuba, Ibaraki, Japan; c Hokkaido Research Station, National Institute of Animal Health, National Agriculture and Food Research Organization, Sapporo, Hokkaido, Japan; d Graduate School of Life and Environmental Sciences, Osaka Prefecture University, Izumisano, Osaka, Japan

## Abstract

*Salmonella enterica* serovar Typhimurium pulsed-field gel electrophoresis cluster VII has been isolated from cattle populations in Japan since the mid-2000s. Some cluster VII isolates exhibited extended-spectrum cephalosporin resistance defined by the *bla*_CMY-2_ gene located in a chromosomal genomic island, GI-VII-6. We determined the whole-genome sequence of strain L-3553 as the reference strain.

## GENOME ANNOUNCEMENT

*Salmonella enterica* serovar Typhimurium strains belonging to pulsed-field gel electrophoresis cluster VII and having no recognized phage type have been dominant among cattle populations in Hokkaido, Japan, since the mid-2000s (1). Some of the cluster VII isolates exhibited resistance to extended-spectrum cephalosporin and harbored a resistance island, GI-VII-6, in their chromosome (2). GI-VII-6 contains multiple antimicrobial resistance genes, including *bla*_CMY-2_. We determined the whole-genome sequence of GI-VII-6-harboring *S*. Typhimurium strain L-3553, which was isolated in 2004, as the reference strain.

The whole-genome sequencing DNA library of this strain was constructed by a Nextera XT DNA sample prep kit (Illumina, San Diego, CA) using an Illumina MiSeq (Illumina) and a MiSeq 500 cycle kit v. 2 (Illumina). The trimmed and filtered short reads were assembled with the *De Novo* Assembler program in CLC Genomics Workbench v. 6.5 (CLC Bio, Aarhus, Denmark). The predicted gaps were amplified using specific PCR primer pairs, followed by Sanger DNA sequencing using a BigDye Terminator v. 3.1 cycle sequencing kit (Applied Biosystems, Foster City, CA). To validate the gap-closed sequences, the short reads were aligned with tentative whole L-3553 genome sequences using BWA-SW (v. 0.6.1) (3) and SAMtools (v. 0.1.18) (4). Gene prediction was performed with the RAST annotation server for the whole-genome sequence (5).

The chromosome of L-3553 comprised 5,051,841 bp (G+C content, 53.18%), and the plasmid denominated as pST3553 comprised 132,611 bp (G+C content, 54.42%). In total, 4,947 and 159 open reading frames were identified in the chromosome and pST3553, respectively. Six prophages were identified in the chromosome, including three common prophages, i.e., Gifsy-1, Gifsy-2, and ST64B, which are present in other *S*. Typhimurium strains. However, the common prophages Fels-1 and Fels-2 were not found. The effector protein-encoding genes *gogB* in Gifsy-1, *sodC* and *sseI* in Gifsy-2, and *sseK3* in ST64B were all intact. Three additional prophage regions were identified using Phage_Finder (6), which were similar to bacteriophages P22 (coverage, 46%; identity, 97%), PsP3 (coverage, 62%; identity, 92%), and P2 (coverage, 72%; identity, 97%). GI-VII-6 contained nine antimicrobial resistance genes, *aadA*, *strA*, *strB*, *bla*_CMY-2_, *floR*, *sul1*, *sul2*, *tet*(A), and *dfrA12*, and was flanked by directly repeated IS*26* copies. No other horizontally acquired antimicrobial resistance genes were observed in the chromosome. The sequence of pST3553 comprised the serovar-specific virulence plasmid pSLT (7) and a single horizontally acquired region containing five antimicrobial resistance genes, including *aadA1*, *aph(3′)-Ic*, *bla*_TEM-1_, *sul1*, and *tet*(A); thus, pST3553 is regarded as a virulence-resistance plasmid. The horizontally acquired region was flanked by IS*1294* copies. The whole sequence of pST3553 was highly similar to that of the pYT2 plasmid (coverage, 100%; identity, 99%), which is harbored by *S*. Typhimurium strain KT262 (8).

### Nucleotide sequence accession numbers.

The whole-genome sequence of *S*. Typhimurium L-3553 has been deposited in the DNA Data Bank of Japan under accession no. AP014565 for the chromosome and AP014566 for the plasmid pST3553.
